# Phanerozoic co-evolution of O_2_-CO_2_ and ocean habitability

**DOI:** 10.1093/nsr/nwae099

**Published:** 2024-03-15

**Authors:** Zunli Lu, Rosalind E M Rickaby, Jonathan L Payne, Ashley N Prow

**Affiliations:** Department of Earth & Environmental Sciences, University, Syracuse, USA; Department of Earth Sciences, University of Oxford, UK; Department of Earth and Planetary Sciences, Stanford University, USA; Department of Earth & Environmental Sciences, University, Syracuse, USA

## Abstract

This perspective reviews how atmospheric compositions, animals and marine algae evolved together to determine global ocean habitability during the past 500 million years.

Atmospheric carbon dioxide and oxygen concentrations are partially linked via the geological cycle of organic carbon (Fig. 1A–C; e.g. CO_2_ + H_2_O ↔ CH_2_O + O_2_). The history of these two biologically active components, controls on their concentrations, and implications for the complexity of the biosphere and habitability of Earth have been hotly debated, but are generally considered independently. Ribulose bisphosphate carboxylase/oxygenase, Rubisco, is the enzyme responsible for all oxygenic photosynthesis, carbon fixation, and is the gatekeeper of energy flow to the animal kingdom. Since Rubisco also fixes O_2_ as part of photorespiration, O_2_ and CO_2_ compete for the active site of Rubisco. Episodes of enhanced organic carbon burial contributed to removing carbon and releasing oxygen to the environment, particularly after the advent of land biota so dramatically increased the O_2_:CO_2_ ratio (Fig. 1B). This increase in O_2_:CO_2_ should have influenced the efficiency of Rubisco, shifting the balance towards the energy-sapping photorespiration and limiting the carbon fixation ability of plants and algae, thereby reducing new productivity and the energy cascade to the higher trophic levels within the ecosystem. However, the complexity of the modern ecosystem has emerged and thrived amidst this backdrop of increasing O_2_:CO_2_ throughout the Phanerozoic, which raises key research questions regarding evolution and habitability. To what extent can the biosphere adapt to variations caused by geological cycles? Are there Gaia-like feedbacks between life and their physical environment that assist in maintaining Earth's habitability? Does the biosphere itself limit the range of environmental possibilities?

**Figure 1.**
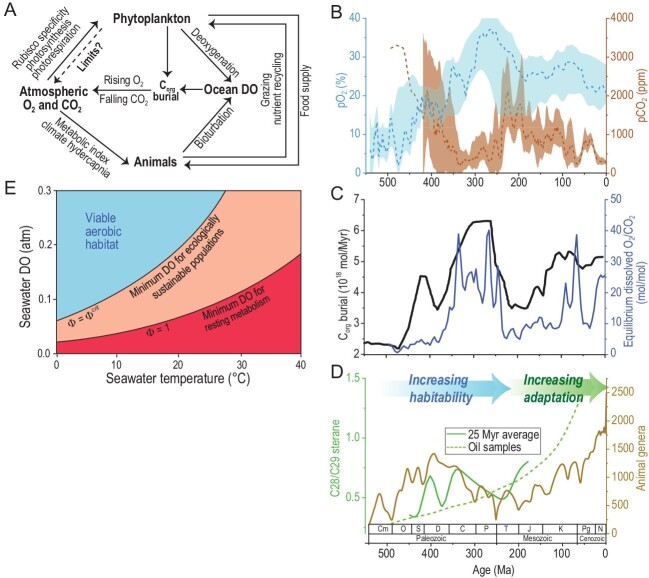
(A) Schematic cartoon illustrating the main processes discussed in this paper. Ocean DO stands for ocean dissolved oxygen. (B) Modelled atmospheric pO_2_ in blue [2]. Proxy-based pCO_2_ estimates in brown solid line, 0–420 Ma [1] and modelled pCO_2_, brown dash line, 420–500 Ma [24] (C) Burial rates of organic matter (black line), compared to the calculated ratio of dissolved O_2_:CO_2_ (blue line) in the ocean using the equations for equilibrium of dissolved CO_2_ and O_2_ concentrations of seawater constrained by temperature and salinity (Supplementary materials). (D) The average C28/C29-sterane ratio of algal biomarkers [10]. The number of genera of marine animals across the Phanerozoic [23]. (E) The range of seawater temperature (°C) and dissolved oxygen (DO) concentrations (atm) for ecological sustainably of a hypothetical ecophysiotype population (modified from [7]) where Φ is its metabolic index defined as the ratio of O_2_ supply to an organism's resting O_2_ demand. The critical metabolic index, Φ^crit^, is the minimal requirement for survival.

Here we link the history of Phanerozoic O_2_ and CO_2_ concentrations and draw together the evolution of marine algal primary producers and the diversity history of marine animals to explore feedbacks between life and the environment. We emphasize that spatially resolved coupled redox and fossil evidence may be key to understanding feedbacks between the biosphere and the geosphere, as well as the drivers and limits on habitability.

## MODEL AND PROXY RECONSTRUCTIONS FOR CO_2_ AND O_2_

Phanerozoic reconstructions of atmospheric pCO_2_ have converged over the last decade (Fig. 1B). Proxy records, such as leaf stomata, pedogenic carbonate δ^13^C and boron isotopes, extend back to ∼420 Ma, showing pCO_2_ peaking above 2000 ppm during two greenhouse episodes (Silurian and early Mesozoic) each followed by declines to near-modern levels associated with icehouse climates [1]. Atmospheric pO_2_ curves derived from mass-balance models agree on low pO_2_ (<∼0.5 PAL) from the Cambrian to early Silurian, in contrast to the rest of the Phanerozoic (1 PAL or higher) [2]. There is disagreement about when pO_2_ reached the highest level (e.g. during the Carboniferous). pO_2_ proxies broadly concur with the modelling [2], although the models based on isotopic mass balance (of δ^13^C and δ^34^S etc.) still have uncertainties. Establishing novel quantitative pO_2_ proxies remains challenging. It is unclear whether the recent pO_2_ proxy estimates are more reliable than the charcoal record, while charcoal production could be influenced by fuel availability for wildfires instead of pO_2_. Overall, the first-order trend is that atmospheric pCO_2_ decreased and pO_2_ increased during the Phanerozoic, albeit with considerable temporal variations and uncertainty.

Climate conditions (reflected in pCO_2_) did not dominate subsurface oceanic O_2_ over the Phanerozoic on the time scale of a hundred million years ([3] and Supplementary materials). Extensive ocean anoxia has been identified in several intervals even under relatively high atmospheric pO_2_ and sometimes associated with major mass extinctions (e.g. [4,5]), highlighting the decoupling between oceanic and atmospheric oxygen levels. Significant spatial heterogeneity in dissolved oxygen (DO) existed in global oceans throughout the Phanerozoic and there is no simple way of predicting temporal changes in the spatial DO pattern [3]. These findings highlight the need to map ocean DO spatially for distinct time slices, regardless of the challenges of DO proxies (Supplementary materials). Earth system models (like cGENIE) are a promising tool to reconcile multiple marine redox proxies with atmospheric composition [6], and produce quantitative global DO estimates critical for constraining extinction vulnerability [7]. A ‘deep-time paleoceanographic data-model comparison’ approach is likely the key to reconstructing Phanerozoic DO patterns, reconciling global and local redox proxy data, and for investigation alongside the evolving biosphere.

## ALGAL EVOLUTION

The oceans experienced three distinctive algal eras, evidenced from three independent sources of microfossils, molecular biomarkers, and molecular clocks for individual clades (e.g. [8]). The ocean was first dominated by cyanobacteria until the end of the Sturtian glaciation, followed by the rise of green algae (Chlorophyta, primary endosymbionts). In the Devonian, there was an expansion of more derived prasinophyte algae (Chlorophyta) [8] before a second major phytoplankton succession took place at the transition from the Palaeozoic to the Mesozoic. At this time, the ocean, dominated by the green Archaeplastida, transformed into one dominated by secondary endosymbiotic algae with red algal-derived plastids, including the haptophytes (e.g. coccolithophores) and heterokont (e.g. diatom) lineages [8–10].

This Phanerozoic algal succession represents selection for more highly discriminant Rubiscos coupled with enhanced obligate aerobic metabolisms [11]. Rising marine O_2_:CO_2_ ratios (Fig. 1C) may have been among the drivers for these different phases of algal domination [10]. The final transition to the secondary endosymbiont bearing red algae lineage may have coincided with a decrease in surface ocean O_2_:CO_2_ (Fig. 1D), but notably a change in the spatial structure of oxygen within the ocean would result in an increased upper ocean oxygen content due to the persistent deepening of the oxygen minimum zones [12].

The compensation points of O_2_ and CO_2_ (Supplementary materials), controlled by the efficiency of photosynthetic pathways, have been proposed to impose absolute limits on atmospheric composition and set the O_2_:CO_2_ of the modern atmosphere [13], although the O_2_-dependency of fire risk may outweigh these biochemical limits. During the Phanerozoic, the terrestrial flora had consistently been dominated by C3 photosynthesis with a Rubisco specificity (τ) of likely ∼80. τ is a unitless measure of the relative affinity and rate of turnover for CO_2_ over O_2_, calculated as τ = (k_cat,C_/K_C_)/(k_cat,O_/K_O_). In the marine realm, the poorly discriminating Precambrian cyanobacterial Rubisco (τ  ∼ 40–50) were surpassed by the intermediate Rubisco of the Chlorophyta (τ  ∼ 60–80) from the Sturtian deglaciation through the Palaeozoic, before the final transition at the Mesozoic to the most highly selective Rubisco of the chlorophyll a + c containing algae (τ ∼ 80–120). O_2_:CO_2_ ratios rose to 5 at ∼400 Ma and then accelerated upwards to persistently high values of 25–40. These inefficient cyanobacteria and green algal Rubiscos would have been pushed close to their carbon compensation point yielding low net carbon fixation rates. Such conditions could have limited the carbon fixation rates for the ecosystem, but promoted the initiation of carbon concentrating mechanisms (e.g. [14]) and enhanced the selective pressure for a more discriminating Rubisco of the red algal lineage. Indeed the emergence of the pyrenoid, an intrachloroplast compartment thought to be adapted to concentrate carbon around Rubisco, in the haptophytes at ∼350 Ma [10] (with positive selection in Rubisco), and in land hornworts ∼100 Ma and <35 Ma [15] all coincide with the highest values of our inferred O_2_:CO_2_ ratio.

Any increase in Rubisco specificity and/or the induction of CO_2_ concentrating mechanisms to elevate chloroplast O_2_:CO_2_ lowers the CO_2_ compensation point and elevates the O_2_ compensation point. Over the Phanerozoic, Rubisco specificity improved by ∼3 fold and the induction of carbon concentrating mechanisms which elevated the internal CO_2_ concentration at the active site of Rubisco, likely enhanced carbon fixation by ∼6–10 fold [16]. As a result of cells harnessing energy to create ancient high CO_2_, low O_2_ conditions at the active site of Rubisco, the CO_2_ compensation point decreased towards the modern, driving a lower habitable CO_2_ concentration. By contrast, even though the O_2_ compensation point is proportional to CO_2_ (which has declined ∼10–20 fold) and was therefore thought to be higher in the past [13], the direct dependence on the Rubisco specificity/carbon fixation efficiency means that the top threshold of habitable O_2_ content of the atmosphere has most likely increased towards its highest value in the modern. The progressive steps of enhanced carbon concentrating efficiency through the Phanerozoic, have permitted higher atmospheric O_2_ and aerobic capacity in the animal kingdom.

## ANIMAL EVOLUTION

Oxygen availability has long been hypothesized as an important control on animal evolution due to its critical role in animal respiration and biosynthesis. More recently, the interaction between oxygen and temperature has been identified as a likely constraint on animal evolution. Metabolic demand in ectothermic animals (to a first approximation, everything that is not a mammal or a bird) increases exponentially with temperature. Consequently, ocean habitability must be considered in terms of the ratio of oxygen supply to oxygen demand (e.g. [7]). An implication of this physiological constraint is that animal tolerance to temperature variation and, especially, to higher temperatures is more limited at lower oxygen concentrations (Fig. 1E). Furthermore, temperature-dependent oxygen deficiency (not holding sufficient oxygen to meet animal metabolic demands) may occur in warm oceans before reaching the hypoxic or anoxic conditions recorded by geochemical proxies [17]. The coupled constraints of low oxygen and warm climate may have limited the earliest animals to deep, cold, thermally stable environments. Some of the earliest motile animals may have burrowed through photosynthetic microbial mats where oxygen produced by local photosynthesis was concentrated [18]. Limited oxygen availability may also have delayed the evolution of predators into the Cambrian due to their greater oxygen demand during prey capture and digestion [19]. Oxygen availability, combined with changes in climate, may also have modulated animal extinction in the oceans across time [20]. The general decline in extinction rates for marine animals across the Palaeozoic (540–252 Mya) has been hypothesized to result from an increase in oxygen availability, providing animals with greater physiological tolerance to changes in climate and greater ability to inhabit productive, shallow-marine environments that can support greater abundance and taxonomic diversity [7] and would have been further supported by overall cooling through this interval. In the Mesozoic, after atmospheric pO_2_ had reached or exceeded present atmospheric levels, oceanic anoxic events, often associated with rapid climate warming pulses, coincided with some mass extinction events (e.g. [4]). Explicit modelling of physiological response to climate warming shows that temperature-dependent hypoxia can explain the spatial gradient in the end-Permian mass extinction [21] and may be useful in predicting the pattern and extent of extinction in the oceans during the next few centuries. Nonetheless, there is less evidence that the ratio of O_2_ to CO_2_ plays the kind of direct and important role in animal physiology and evolution that it does for algae and plants (Supplementary materials), although the haemoglobin and haemocyanin binding affinity for O_2_ is diminished under elevated CO_2_ conditions (the Bohr effect).

## CO-EVOLUTION OF THE PHYSICAL ENVIRONMENT AND BIOSPHERE

The general cooling of our planet via a first-order decline of pCO_2_ and the contrasting rise of the oxygen content accompanied two phases in the changing habitability for photosynthetic algae and animals: (1) the initial increase in marine habitability and (2) the subsequent biological adaptation/innovation as the atmospheric composition started to impinge on the opposite end of their physiological comfort zone (Fig. 1D). The evolution and advancement of the carbon concentrating mechanism might have been an essential step in the atmospheric engineering of the photosynthesizers to enable ever diminishing pCO_2_ whilst allowing atmospheric pO_2_ to further increase, maintaining a cooler and more oxygen-rich environment for the animals. Animals with closed circulatory systems, air-breathing (better access to O_2_), greater levels of activity, and more control of body temperature are increasingly diverse and successful, becoming more independent of external conditions over time [22]. Both phytoplankton and animals are operating further from their natural limits over time, using energy to control the chemistry of their cellular environments to decouple their metabolisms from the environment, even if the environment itself would be less favourable had the organisms not evolved.

Associated with each algal transition is an increase in cell sizes of the phytoplankton, allowing greater compartmentalization and internal control, the addition of mineralizing skeletons which propagated intermediate-depth oxygenation [12] and accelerated the transfer of primary productivity towards larger-size organisms and higher trophic levels [9]. These transitions in the dominant groups of phytoplankton, each of which may have expanded the effective base of the food chain relative to the last, may help explain the long-term increases in the taxonomic diversity and ecological complexity (e.g. [23]). Such increases in animal size, motility, and levels of bioturbation may have recycled nutrients for marine photosynthesizers more efficiently and thus further stabilized biogeochemical cycles (Fig. 1A).

Future breakthroughs in understanding the co-evolution of atmospheric composition and Earth habitability may emerge from the ‘triple-junction’ of spatially resolved records of (1) ocean oxygen concentrations, (2) algal photosynthesis and associated biomarker evidence, and (3) quantitative estimates of animal metabolic tolerance and their corresponding fossil records.

## Supplementary Material

nwae099_Supplemental_File
