# Supplementary material for: Phanerozoic co-evolution of O2-CO2 and ocean habitability
Source: Natl Sci Rev. 2024 Mar 15;11(6):nwae099. doi: 10.1093/nsr/nwae099 (PMC11194836; doi:10.1093/nsr/nwae099)
Supplement: nwae099_Supplemental_File [file nwae099_supplemental_file.docx]

**Supplementary materials**

**O_2_/CO_2_**

O_2_/CO_2_ in the ocean were calculated with the equations for equilibrium dissolved CO_2_ and O_2_ concentrations of seawater constrained by temperature and salinity (Benson & Krause,1984; Weiss, 1974). Phanerozoic temperature and salinity data are from Hay et al., 2006; Grossman and Joachimski 2022. This calculation does not capture the spatial variability at any particular time slice.

**Oceanic oxygen concentrations and their proxies**

Even though ocean deoxygenation has been associated with anthropogenic warming over decadal time scales (Oschlies et al., 2018; Breitburg et al., 2018), global temperature is not the primary control of subsurface oceanic O_2_ over the Phanerozoic. Foraminiferal assemblages suggest that Pliocene ocean circulation may have induced a spatial pattern of oxygen minima distinct from the modern ocean, despite the similar pCO_2_ to modern day (Davis et al., 2023). There is increasing evidence for episodes of well-oxygenated oceans during warm periods (Auderset et al., 2022; Hess et al., 2023) and deoxygenation during cold periods (e.g., Bartlett et al., 2018).

Marine habitats, especially on the continental margins, have been important for life throughout the Phanerozoic and shallow-ocean conditions have been assumed to represent equilibrium with the atmosphere (e.g., Holland 2006). Many marine redox proxies and corresponding box models (e.g., δ^238^U, ε^205^Tl, δ^34^S) estimate the global extent of anoxia without pinpointing the locations of anoxia and the degree of spatial variability (Zhang et al., 2020; Them et al., 2018; He et al., 2020). Other local proxies applicable in deep time, such as Fe speciation (e.g., Sperling et al., 2021) and I/Ca (Lu et al., 2018), do not translate easily or directly into quantitative dissolved O_2_ (DO) values, although the I/Ca threshold values have been calibrated empirically to DO levels based on studying recent planktic and benthic foraminifera shells (Lu et al., 2016; Lu et al., 2020; Lu et al., 2020). Instead of finding one perfect quantitative proxy, multi-proxy data model comparison is likely the key to better constraining ocean DO.

**The compensation points of O_2_ and CO_2_**

An oxygen compensation point exists (for a given CO_2_) at which oxygen evolution from photosynthesis is matched by photooxidation and above which plant/algal growth should not be possible (Berry et al., 1994; Tolbert et al., 1995). The CO_2_ compensation point is the CO_2_ concentration at which net CO_2_ fixation is zero at a given O_2_ level and temperature. These compensation points depend on both the ratio of CO_2_ and O_2_ concentrations in the chloroplast and the specificity of Rubisco. According to the biochemical stoichiometry of carbon fixation, at the CO_2_ compensation point, the rate of carboxylation is 0.5 that of oxygenation so that: vc/vo= 0.5 = τ [CO_2_]/[O_2_] where τ is Rubisco specificity. The CO_2_ compensation point is proportional to [O_2_] but inversely proportional to Rubisco specificity/CCM (carbon concentrating mechanism) efficiency.

**pCO_2_, O_2_/CO_2_, and animal physiology**

Direct physiological impacts of increased pCO_2_ have been hypothesized less commonly. Knoll et al. (1996) suggested that hypercapnia could have caused the distinctive taxonomic selectivity of the end-Permian mass extinction and numerous subsequent studies have suggested that ocean acidification and reduced carbonate saturation, indirectly related to atmospheric pCO_2_ (Hönisch et al., 2012), may have contributed to the end-Permian, end-Triassic, and other extinction events (e.g., Knoll et al., 2007; Hautmann 2004, 2008; Kiessling and Simpson, 2011). pH affects oxygen binding affinity of oxygen binding proteins, so CO_2_ can have indirect effects on oxygen delivery in animals (Seibel and Childress, 2013). It is unlikely that the ratio of O_2_ to CO_2_ plays the kind of direct role in animal physiology that it does for algae and plants. Thermodynamics strongly favour the aerobic oxidation of organic matter that forms the foundation of animal metabolism under all realistic conditions that would support aerobic animals to begin with (Seibel and Childress, 2013). Physiological experiments likewise indicate a synergistic interaction between warming and deoxygenation but not for pCO_2_ or pH with other factors (Reddin et al., 2020).

**Reference**

Auderset, A., Moretti, S., Ebner, P., Kast, E., Wang, X. T., Schiebel, R., et al. (2022). Enhanced ocean oxygenation during Cenozoic warm periods. *Nature*, 609. https://doi.org/https://doi.org/10.1038/s41586-022-05017-0

Bartlett, R., Elrick, M., Wheeley, J. R., Polyak, V., Desrochers, A., & Asmerom, Y. (2018). Abrupt global-ocean anoxia during the Late Ordovician–early Silurian detected using uranium isotopes of marine carbonates. *Proceedings of the National Academy of Sciences of the United States of America*, 115(23), 5896–5901. https://doi.org/10.1073/pnas.1802438115

Benson, B. B., & Krause Jr, D. (1984). The concentration and isotopic fractionation of oxygen dissolved in freshwater and seawater in equilibrium with the atmosphere. *Limnology and oceanography*, 29(3), 620-632.

Berry, J. A., Collatz, G. J., Guy, R. D., & Fogel, M. D. (1994). The compensation point: can a physiological concept be applied to global cycles of carbon and oxygen. Regulation of Atmospheric CO2 and O2 by Photosynthetic Carbon Metabolism, Oxford Univ. Press, New York, 234-238.

Breitburg, D., Levin, L. A., Oschlies, A., Grégoire, M., Chavez, F. P., Conley, D. J., Garçon, V., Gilbert, D., Gutiérrez, D., Isensee, K., Jacinto, G. S., Limburg, K. E., Montes, I., Naqvi, S. W. A., Pitcher, G. C., Rabalais, N. N., Roman, M. R., Rose, K. A., Seibel, B. A., & Telszewski, M. (2018). Declining oxygen in the global ocean and coastal waters. *Science*, 359(6371). https://doi.org/10.1126/science.aam7240

Davis, C. V., Sibert, E. C., Jacobs, P. H., Burls, N., & Hull, P. M. (2023). Intermediate water circulation drives distribution of Pliocene Oxygen Minimum Zones. *Nature Communications*, 14(1), 1–11. https://doi.org/10.1038/s41467-022-35083-x

Grossman, E. L., & Joachimski, M. M. (2022). Ocean temperatures through the Phanerozoic reassessed. Scientific Reports, 12(1). https://doi.org/10.1038/s41598-022-11493-1

Hay, W. W., Migdisov, A., Balukhovsky, A. N., Wold, C. N., Flögel, S., & Söding, E. (2006). Evaporites and the salinity of the ocean during the Phanerozoic: Implications for climate, ocean circulation and life. *Palaeogeography, Palaeoclimatology, Palaeoecology*, 240(1-2), 3–46. https://doi.org/10.1016/j.palaeo.2006.03.044

Hautmann, M. (2004). Effect of end-Triassic CO2 maximum on carbonate sedimentation and marine mass extinction. *Facies*, 50(2), 257–261. https://doi.org/10.1007/s10347-004-0020-y

Hautmann, M., Benton, M. J., & Tomašových, A. (2008). Catastrophic ocean acidification at the Triassic-Jurassic boundary. *Neues Jahrbuch Fur Geologie Und Palaontologie - Abhandlungen*, 249(1), 119–127. https://doi.org/10.1127/0077-7749/2008/0249-0119

He, T., Dal Corso, J., Newton, R. J., Wignall, P. B., Mills, B. J. W., Todaro, S., et al. (2020). An enormous sulfur isotope excursion indicates marine anoxia during the end-Triassic mass extinction. *Science Advances*, 6(37), 2–10. https://doi.org/10.1126/sciadv.abb6704

Hess, A. V., Auderset, A., Rosenthal, Y., Miller, K. G., Zhou, X., Sigman, D. M., & Martínez‐García, A. (2023). A well-oxygenated eastern tropical Pacific during the warm Miocene. *Nature*, 619(7970), 521–525. https://doi.org/10.1038/s41586-023-06104-6

Holland, H. D. (2006). The oxygenation of the atmosphere and oceans. *Philosophical Transactions of the Royal Society B: Biological Sciences*, 361(1470), 903–915. https://doi.org/10.1098/rstb.2006.1838

Hönisch, B., Ridgwell, A., Schmidt, D. N., Thomas, E., Gibbs, S. J., Sluijs, A., et al. (2012). The geological record of ocean acidification. *Science*, 335(6072), 1058–1063. https://doi.org/10.1126/science.1208277

Kiessling, W., & Simpson, C. (2011). On the potential for ocean acidification to be a general cause of ancient reef crises. *Global Change Biology*, 17(1), 56–67. https://doi.org/10.1111/j.1365-2486.2010.02204.x

Knoll, A. H., Bambach, R. K., Canfield, D. E., & Grotzinger, J. P. (1996). Comparative earth history and late Permian mass extinction. *Science*, 273(5274), 452–457. https://doi.org/10.1126/science.273.5274.452

Knoll, A. H., Bambach, R. K., Payne, J. L., Pruss, S., & Fischer, W. W. (2007). Paleophysiology and end-Permian mass extinction. *Earth and Planetary Science Letters*, 256(3-4), 295-313.

Lu, W., Ridgwell, A., Thomas, E., Hardisty, D. S., Luo, G., Algeo, T. J., et al. (2018). Late inception of a resiliently oxygenated upper ocean. *Science*, 177(July), 174–177. doi/10.1126/science.aar5372

Lu, W., Dickson, A. J., Thomas, E., Rickaby, R. E. M., Chapman, P., & Lu, Z. (2020). Refining the planktic foraminiferal I/Ca proxy: Results from the Southeast Atlantic Ocean. *Geochimica et Cosmochimica Acta,* 287, 318–327. https://doi.org/10.1016/j.gca.2019.10.025

Lu, Z., Hoogakker, B. A. A., Hillenbrand, C., Zhou, X., Thomas, E., Gutchess, K. M., et al. (2016). Oxygen depletion recorded in upper waters of the glacial Southern Ocean, *Nature Communications .* https://doi.org/10.1038/pj.2016.37

Lu, Z., Lu, W., Rickaby, R. E. M., & Thomas, E. (2020). Earth History of Oxygen and the iprOxy. Cambridge Elements. Cambridge: Cambridge University Press. https://doi.org/10.1017/9781108688604

Oschlies, A., Brandt, P., Stramma, L., & Schmidtko, S. (2018). Drivers and mechanisms of ocean deoxygenation. *Nature Geoscience*, 11(7), 467–473. https://doi.org/10.1038/s41561-018-0152-2

Reddin, C. J., Nätscher, P. S., Kocsis, Á. T., Pörtner, H. O., & Kiessling, W. (2020). Marine clade sensitivities to climate change conform across timescales. *Nature Climate Change*, 10(3), 249-253.

Seibel, B. A., & Childress, J. J. (2013). The real limits to marine life: a further critique of the Respiration Index. *Biogeosciences,* 10(5), 2815–2819. https://doi.org/10.5194/bg-10-2815-2013

Sperling, E. A., Melchin, M. J., Fraser, T., Stockey, R. G., Farrell, U. C., Bhajan, L., Brunoir, T. N., Cole, D. B., Gill, B. C., Lenz, A., Loydell, D. K., Malinowski, J., Miller, A. J., Plaza-Torres, S., Bock, B., Rooney, A. D., Tecklenburg, S. A., Vogel, J. M., Planavsky, N. J., & Strauss, J. V. (2021). A long-term record of early to mid-Paleozoic marine redox change. *Science Advances*, 7(28). https://doi.org/10.1126/sciadv.abf4382

Them, T. R., Gill, B. C., Caruthers, A. H., Gerhardt, A. M., Gröcke, D. R., Lyons, T. W., et al. (2018). Thallium isotopes reveal protracted anoxia during the Toarcian (Early Jurassic) associated with volcanism, carbon burial, and mass extinction. *Proceedings of the National Academy of Sciences*, 115(26), 6596–6601. https://doi.org/10.1073/pnas.1803478115

Tolbert, N. E., Benker, C., & Beck, E. (1995). The oxygen and carbon dioxide compensation points of C3 plants: Possible role in regulating atmospheric oxygen. *Proceedings of the National Academy of Sciences of the United States of America*, 92(24), 11230–11233. https://doi.org/10.1073/pnas.92.24.11230

Weiss, R. (1974). Carbon dioxide in water and seawater: the solubility of a non-ideal gas. *Marine chemistry*, 2(3), 203-215.

Zhang, Q., Bendif, E. M., Zhou, Y., Nevado, B., Shafiee, R., & Rickaby, R. E. M. (2022). Declining metal availability in the Mesozoic seawater reflected in phytoplankton succession. *Nature Geoscience*, 15(11), 932–941. https://doi.org/10.1038/s41561-022-01053-7
